# Multiple chemical scaffolds inhibit a promising *Leishmania* drug target

**DOI:** 10.1107/S2052252514014572

**Published:** 2014-06-24

**Authors:** Malcolm Walkinshaw

**Affiliations:** a Centre for Translational and Chemical Biology, School of Biological Sciences, University of Edinburgh, Mayfield Road, Edinburgh EH9 3JR, United Kingdom

**Keywords:** *N*-myristoyltransferase, inhibitor, ligand binding, Leishmania, drug discovery

## Abstract

The need for an effective oral therapy for leishmaniasis is addressed through the study of the target *N*-myristoyltransferase from *Leishmania major*.

Protozoan parasites belonging to the genera *Leishmania* and *Trypanosoma* of the Trypanosomatidae family cause a variety of life threatening and debilitating diseases including kala-azar (the visceral form of leishmaniasis), sleeping sickness (Human African Trypanosomiasis, or HAT) and Chagas disease (American Trypanosomiasis). It is over one hundred years since Paul Ehrlich first coined the term ‘chemotherapy’ and managed to cure trypanosome infections in mice by injecting them with synthetic dye-like molecules including trypan red and trypan blue (Ehrlich, 1907 ▶). Many of the drugs like suramin and melarsoprol that were developed on the back of Ehrlich’s work are still being used to treat these diseases today. However, most of the treatments for leishman­iasis and trypanosomiasis have severe side effects and frequently require to be given intravenously. There is therefore still a very real need for effective oral therapies that can be given without hospitalizing the patient.

The lack of a commercial incentive for the pharmaceutical industry has been one reason that few new drugs have emerged since the 1950s (Barrett & Croft, 2012 ▶). However, over the last few years there has been a strong resurgence of interest in structure-based and target-based studies on these trypanosomatid diseases, led mainly by academic groups – but frequently with expert support from the once-dormant pharmaceutical companies. Indeed the structure-based study led by the York/London groups described in this issue (Brannigan *et al.*, 2014 ▶) was initiated by hits from a high-throughput screen of 150 000 compounds made available by Pfizer (Bell *et al.*, 2012 ▶). There is now a major initiative involving companies including GSK, Roche and Novartis to work with the WHO and other charitable organizations to control and, if possible, eradicate many of the 17 identified Neglected Tropical Diseases including leishmaniasis by 2020 (http://unitingtocombatntds.org/vision-mission).

An important and still relatively untapped resource leading to the identification of specific drug targets against leishmaniasis and other trypanosomatid diseases is the available data from the sequenced parasite genomes. Information from the literature and various genetic and RNAi screens has been compiled into a database listing potential protein targets and summarizing their properties to give an idea of how ‘druggable’ they may be (http://tdrtargets.org/). About 1500 of the 9000 genes in *Leishmania* are classified as essential, suggesting that if the gene is disrupted or severely knocked down the parasite will die. These are clearly the best targets to go after with the hope that a specific inhibitor binding to the protein will mimic the knock-down of the gene expression and kill the parasite. Another desirable property for a drug target is uniqueness – particularly for infectious diseases where the host may lack a gene that is essential to the parasite.

The target *N*-myristoyltransferase from *Leishmania major* studied in work described by Tony Wilkinson and colleagues (Brannigan *et al.*, 2014 ▶) meets this first criterion as LmNMT is essential to a number of parasites. However, NMT is by no means unique to the parasite and is present in all eukaryotic organisms. The job of this enzyme is to transfer the 14-carbon-long fatty acid, myristate, from CoA onto the N-terminal glycine of selected proteins. This post-translational modification is used to anchor the protein into membranes within the cell. Gene disruption has been used to show that the NMT gene is essential in both *Leishmania donovani* (which causes visceral leishmaniasis) and *Leishmania major* (which causes cutaneous leishmaniasis). Earlier work on NMT from the sleeping sickness parasite, *Trypanosoma brucei* and the malarial protozoan parasite *Plasmodium falciparum*, also showed that the organisms died when the enzyme was inhibited or knocked-down. In work led from Dundee that also involved the York group, a series of low nanomolar inhibitors against TbNMT were identified that killed parasites very effectively with ED_50_ values less than 5 n*M* (Frearson *et al.*, 2010 ▶).

The major snag with such potent compounds in the HAT project was that they did not cross the blood brain barrier. In addition, there was a very poor (twofold) specificity for inhibition by the lead compound of TbNMT over the human enzyme (usually a target factor of approaching 100 would be desirable). In fact, the low specificity was found not to be a problem in cellular assays as the prodigious endocytotic activity makes *T. brucei* far more sensitive to NMT than human cells where no toxicity is observed. For HAT infection it is crucial that the drug crosses the brain barrier as tryp­ano­somes invade the brain in the fatal stages of the disease; however, brain penetration is not an issue for leishmaniasis as the parasites take up residence inside human macrophages – providing themselves with another layer of protection from both drugs and the mammalian immune system.

The four inhibitor X-ray structures presented in the York/London paper have IC_50_ values from 1.2 µ*M* down to 30 n*M*. As might be expected, there is little difference between inhibition of the LmNMT and LdNMT values as the two enzymes only differ by 11 amino acids out of 421. Despite these minimal differences it was apparently impossible to obtain crystals of *L. donovani* NMT complexed with inhibitors and all crystallographic work was carried out using the *L. major* protein.

All crystal structures presented in this work are loaded with CoA-myristate in the active site and the inhibitors all bind in the adjacent substrate binding pocket (Fig. 1 ▶). There are remarkably few changes in protein structure upon ligand binding; the only notable feature being alternative conformations of the side chains of Tyr217 and His219. Despite the different chemical scaffolds, all compounds manage to squeeze into the substrate binding pocket burying over 80% their accessible surface area. Most also hydrogen bond with the catalytically important terminal carboxyl group of Leu421 (buried and out of site at the bottom of the substrate pocket in Fig. 1 ▶) as well as the side chain of a tyrosine at the top of the pocket. Encouragingly the four diverse ligands show reasonable specificity of between 10 and 200, over human NMT. This contrasts with the poor specificity but much enhanced binding of DDD646 which inhibits Tb, Ld and Lm NMT all with single-digit nanomolar affinities, but also inhibits the human enzyme almost equally well. There is no obvious structural explanation for the better specificities of the York/London inhibitors, as the binding site is highly conserved between the parasite and human structures – though there is hint that the orientation of the side chain of Tyr217 (highlighted in Fig. 1 ▶) may play a role. Compounds that show better specificity seem to have a preference for out-facing conformations of Tyr217 in the LmNMT structures while those inhibitors (like DDD646, Fig. 1 ▶) which allow Tyr217 to swing in are less specific (but can bind very tightly).

Specificity may well be one of the key issues in taking these compounds onto the next stage of selecting a preclinical anti-leishmania lead from these new scaffolds, and these structures provide an excellent starting point for the next wave of medicinal chemistry to try and enhance both potency and specificity.

## Figures and Tables

**Figure 1:**
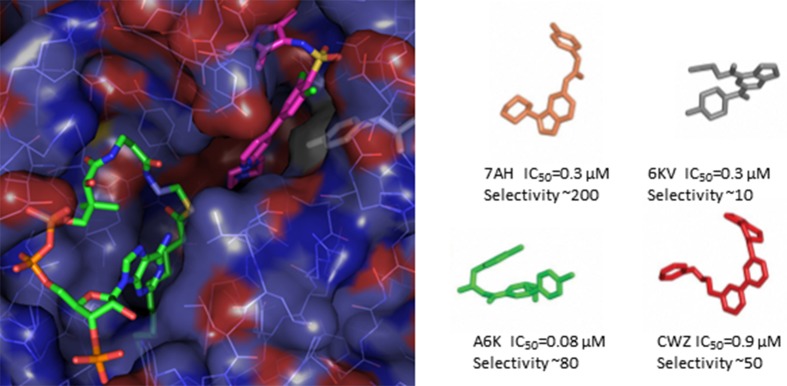
Left: the X-ray structure of LmNMT (2wsa) in complex with DDD646 (magenta C atoms) occupying the substrate binding site. The adjacent active site is occupied by myristoyl-CoA (green C atoms). Tyr217 is highlighted in white. Right: the four ligands complexed with LmNMT described in the current paper and also found to occupy the substrate binding pocket.
